# Supplementary material for: RNF135 Expression Marks Chemokine (C-C Motif) Ligand-Enriched Macrophage–Tumor Interactions in the Glioblastoma Microenvironment
Source: Cancers (Basel). 2025 Oct 9;17(19):3271. doi: 10.3390/cancers17193271 (PMC12523845; doi:10.3390/cancers17193271)
Supplement: Supplementary file 1 [file cancers-17-03271-s001.zip › cancers-3872023-supplementary.pdf]

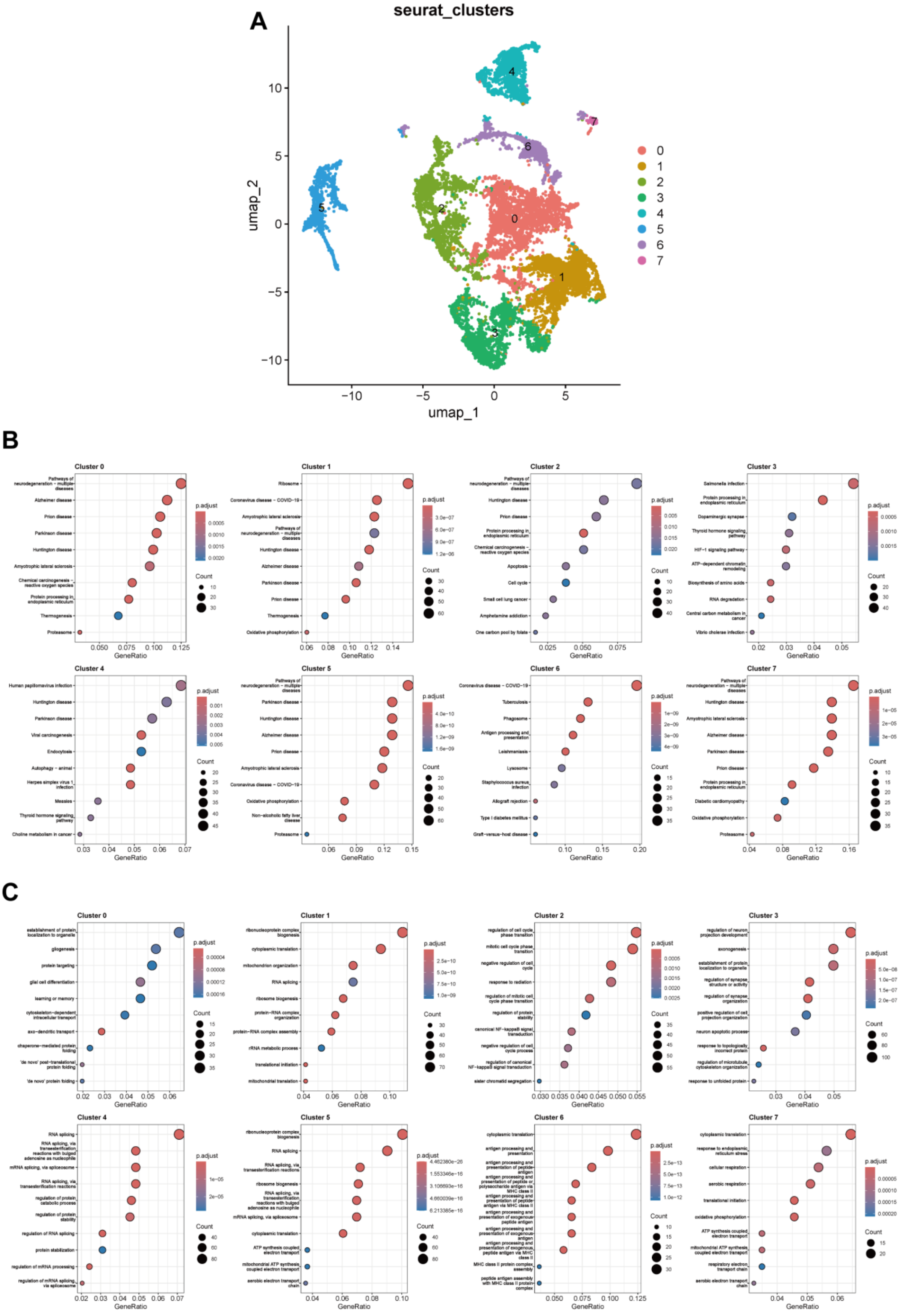

Supplementary Figure S1. Clustering and functional annotation of malignant tumor cell subsets

in GBM.

(A) UMAP visualization of unsupervised clustering results identifies eight distinct malignant cell clusters (Clusters 0–7).

(B–C) GO (B) and KEGG (C) enrichment analyses of each cluster reveal functionally diverse transcriptional programs, including cell cycle regulation, hypoxia response, interferon signaling, and extracellular matrix remodeling.

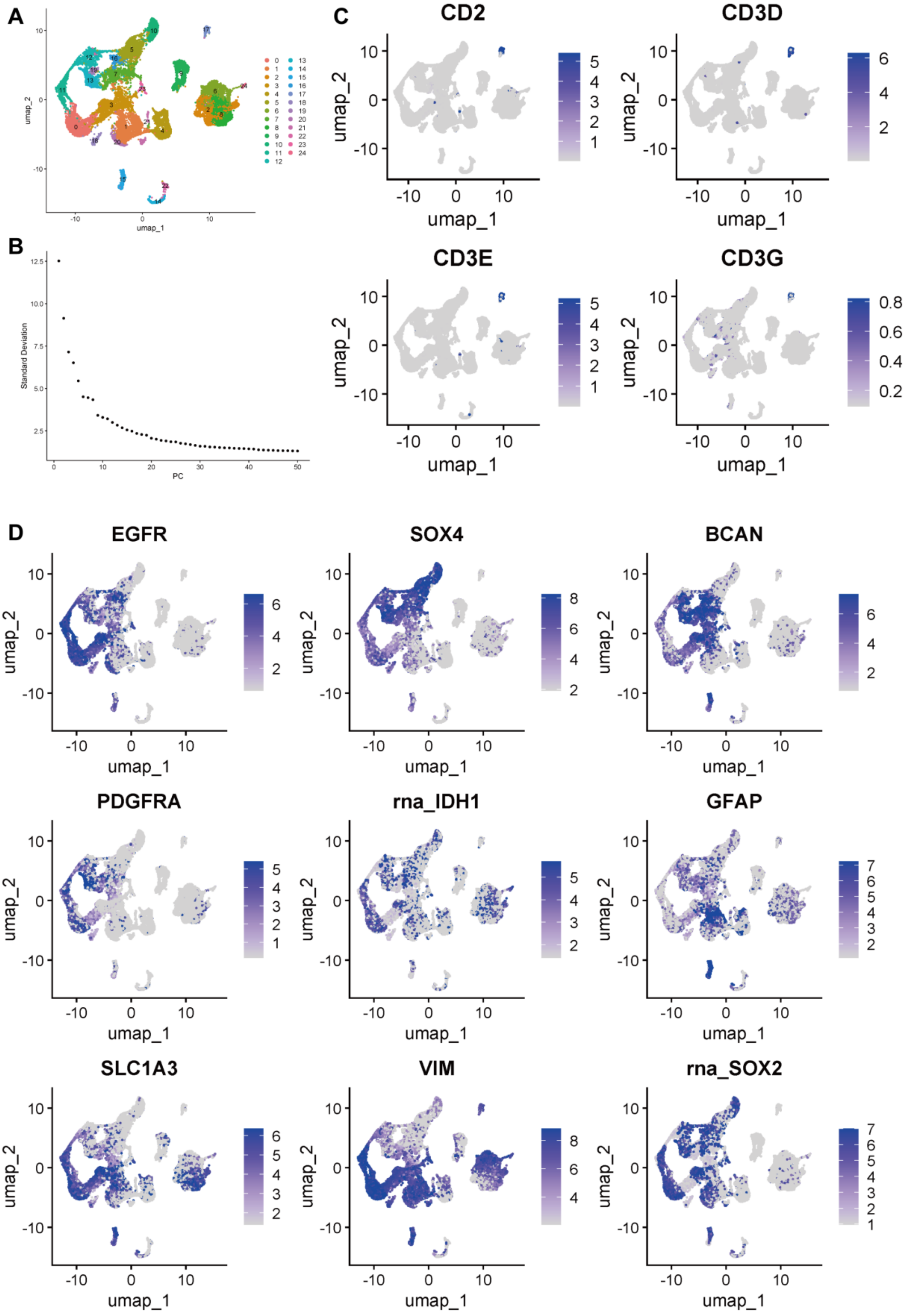

Supplementary Figure S2. Dimensional reduction and marker validation of GBM single-cell data.

(A) UMAP plot displaying individual cell clusters derived from single-cell transcriptomic profiling of GBM.
(B) Elbow plot of principal components showing variance explained to determine optimal dimensions for downstream analysis.
(C) Feature plots showing the expression of T cell markers (*CD2, CD3D, CD3E, CD3G*) across UMAP, used to define T cell populations.
(D) Expression of canonical tumor cell markers.

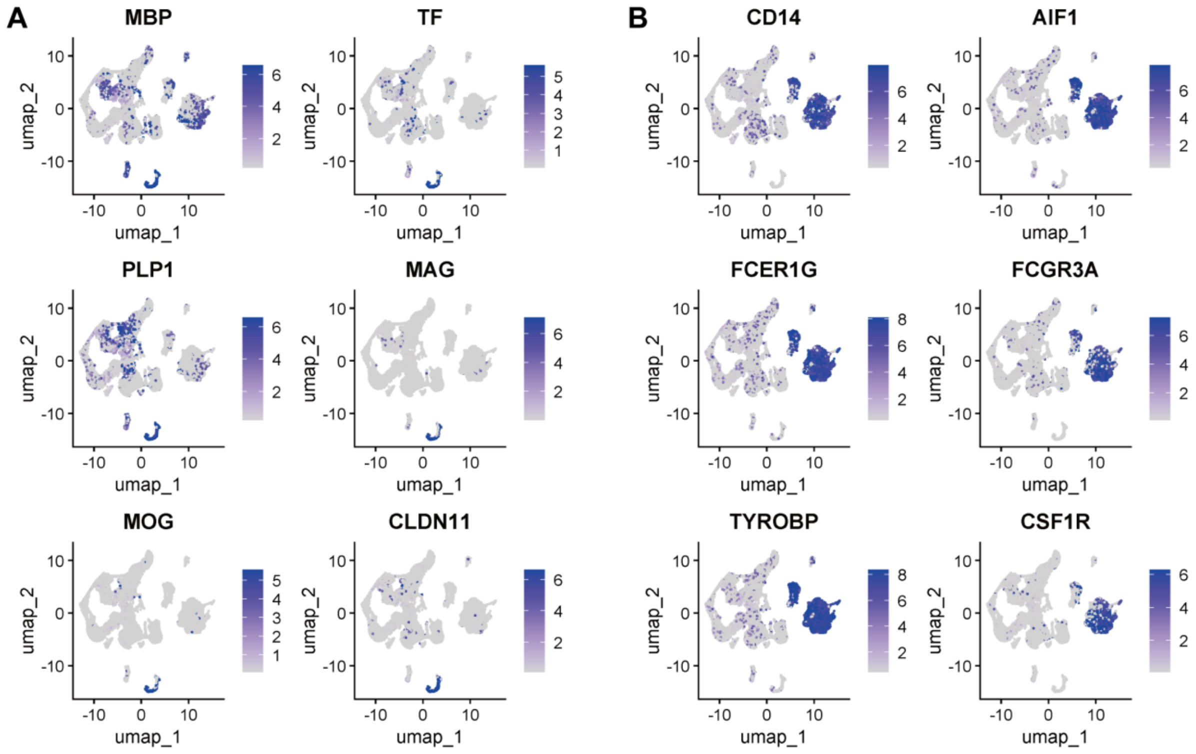

Supplementary Figure S3. Marker gene expression patterns for annotation of oligodendrocyte, and macrophage clusters.

(A) Feature plots of oligodendrocyte-related genes (*MBP, TF, PLP1, MAG, MOG, CLDN11*) showing specific expression in corresponding clusters.

(B) Feature plots of macrophage-related genes (*CD14, AIF1, FCER1G, FCGR3A, TYROBP, CSF1R*) confirming macrophage identity of a major cell cluster.

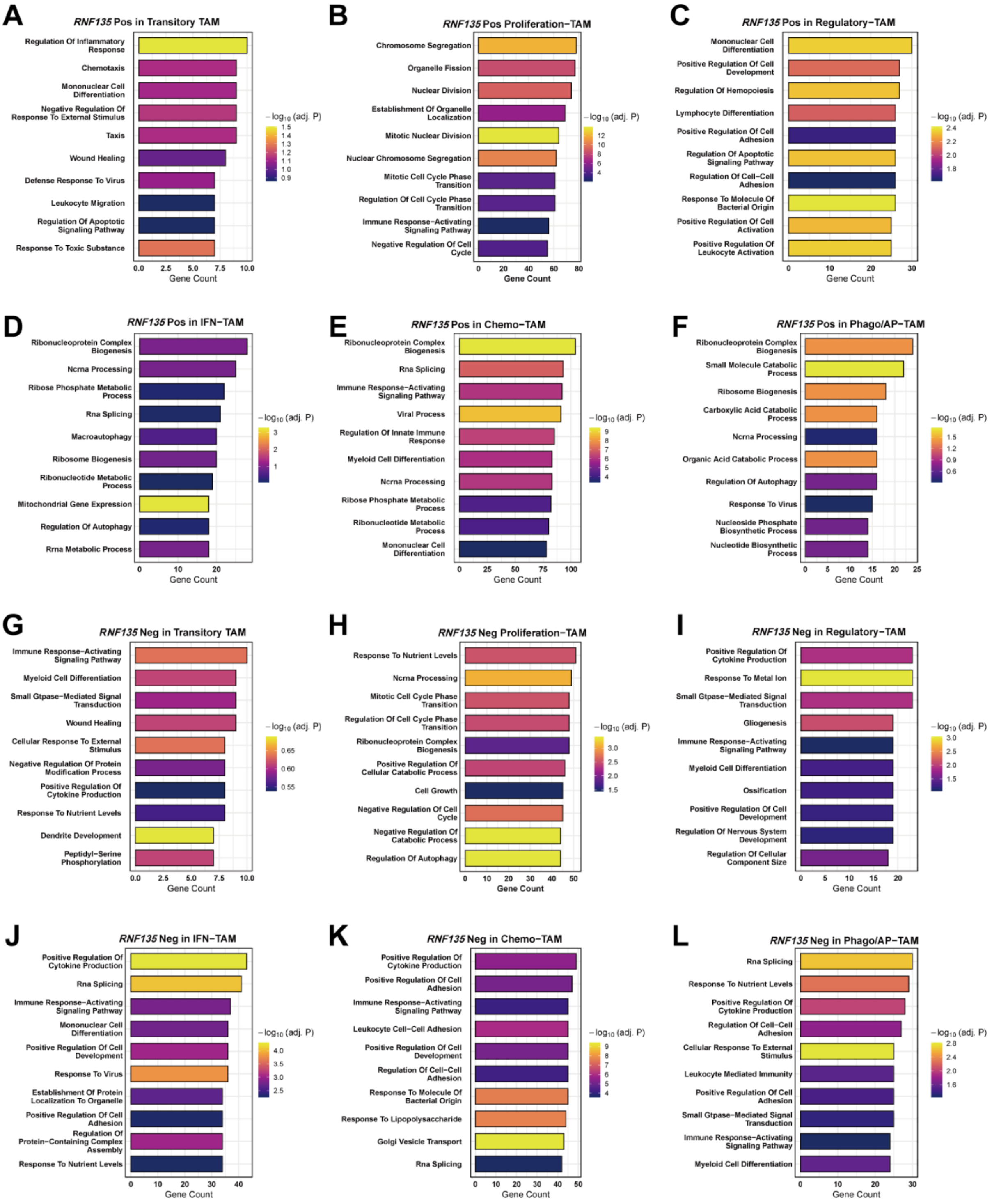

Supplementary Figure S4. GO enrichment analysis of *RNF135*-positive and *RNF135*-negative TAM subsets.

(A–F) GO enrichment for *RNF135*-positive TAM subtypes:

(A) Transitory-TAMs were enriched in inflammatory and wound response pathways;

(B) Proliferation-TAMs in mitotic cell cycle and chromosome segregation;

(C) Regulatory-TAMs in immune regulation and hematopoiesis;

(D) IFN-TAMs in RNA metabolism and ribonucleoprotein complex biogenesis;

(E) Chemo-TAMs in RNA processing and intracellular transport;

(F) Phago/AP-TAMs in metabolism and autophagy-related processes.

(G–L) GO enrichment for *RNF135*-negative counterparts:

(G) Transitory-TAMs showed activation of stress response and small GTPase-mediated signaling;

(H) Proliferation-TAMs were associated with nutrient sensing and autophagy;

(I) Regulatory-TAMs were enriched in cytokine signaling and complement regulation;

(J) IFN-TAMs in cytokine activation and antigen presentation;

(K) Chemo-TAMs in splicing, response to bacteria, and leukocyte activation;

(L) Phago/AP-TAMs in phagocytosis and differentiation processes.

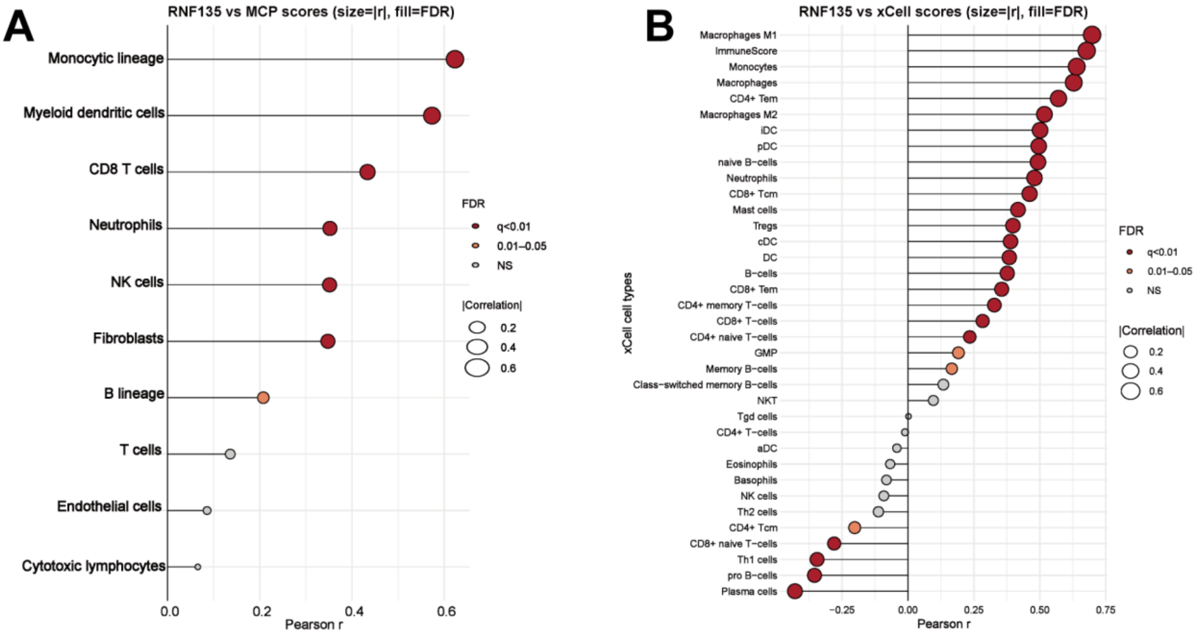

Supplementary Figure 5. Correlation of RNF135 expression with immune and stromal cell populations estimated by MCP-counter and xCell.

(A) Bubble plot showing the Pearson correlations between RNF135 expression and immune cell abundance scores inferred by MCP-counter. Each bubble represents one cell population, with the x-axis showing correlation coefficients (r), bubble size indicating |r|, and bubble color denoting false discovery rate (FDR).

(B) Bubble plot showing the Pearson correlations between RNF135 expression and immune cell populations estimated by xCell. Similar to panel A, the x-axis indicates Pearson r, bubble size reflects |r|, and bubble color corresponds to FDR.
